# A comparison of the cost-effectiveness of treatment of prolonged acute convulsive epileptic seizures in children across Europe

**DOI:** 10.1186/s13561-014-0006-6

**Published:** 2014-04-12

**Authors:** Dawn C Lee, Daniel Gladwell, Anthony J Hatswell, Joshua Porter, Nic Brereton, Elaine Tate, Alison L Saunders

**Affiliations:** 1 BresMed, North Church House, 84 Queen Street, Sheffield S1 2DW, UK; 2 BioExcel, Cornbury Park, Charlbury, Oxfordshire OX7 3EW, UK

**Keywords:** Epilepsy, Cost–utility modelling, BUCCOLAM, Health technology assessment

## Abstract

In the majority of children and adolescents with epilepsy, optimal drug therapy adequately controls their condition. However, among the remaining patients who are still uncontrolled despite mono-, bi- or tri-therapy with chronic anti-epileptic treatment, a rescue medication is required. In Western Europe, the licensed medications available for first-line treatment of prolonged acute convulsive seizures (PACS) vary widely, and so comparators for clinical and economic evaluation are not consistent. No European guidelines currently exist for the treatment of PACS in children and adolescents and limited evidence is available for the effectiveness of treatments in the community setting. The authors present cost-effectiveness data for BUCCOLAM® (midazolam oromucosal solution) for the treatment of PACS in children and adolescents in the context of the treatment pathway in seven European countries in patients from 6 months to 18 years. For each country, the health economic model consisted of a decision tree, with decision nodes informed by clinical data and expert opinion obtained via a Delphi methodology. The events modelled are those associated with a patient experiencing a seizure in the community setting. The model assessed the likelihood of medication being administered successfully and of seizure cessation. The associated resource use was also modelled, and ambulance call-outs and hospitalisations were considered. The patient’s quality of life was estimated by clinicians, who completed a five-level EuroQol five dimensions questionnaire from the perspective of a child or adolescent suffering a seizure. Despite differences in current therapy, treatment patterns and healthcare costs in all countries assessed, BUCCOLAM was shown to be cost saving and offered increased health-related benefits for patients in the treatment of PACS compared with the current local standard of care.

## Background

Even in a relatively homogeneous region such as Western Europe, with similar population demographics and state-provided healthcare systems, differences in culture, legislation and financial incentives can mean that the treatment of patients with the same condition varies between countries. In some disease areas, the pathway of care (even the healthcare setting; that is, primary versus secondary care) may, therefore, differ between countries, and some drugs may be used routinely in one country but not be available in another. A consequence of these variations is that pharmaceutical companies are faced with different challenges in persuading health technology assessment (HTA) agencies in each country of the value of their product. In this paper, we consider the example of BUCCOLAM (midazolam oromucosal solution; available from ViroPharma SPRL – BVBA, Belgium) in the treatment of prolonged, acute, convulsive seizures (PACS) in children and adolescents [1].

Across Europe, 130,000 new cases of epilepsy are recorded each year among children and adolescents (an incidence rate of 70–80 per 100,000) [2,3]. The incidence is particularly high during the first year of life, and the likelihood of developing the condition then decreases during childhood [3]. Anti-epileptic drug therapy is the primary treatment for children with epilepsy, with the aim of preventing seizures [4], and approximately 70% of patients become seizure-free with optimal drug therapy [3,5]. However, patients do not always receive optimal drug therapy and approximately 50–60% of patients with epilepsy experience breakthrough seizures during the course of a year, some of whom will require a prescription for rescue medication [2]. Children who have severe, symptomatic epilepsy are those who are most commonly prescribed rescue medication [6].

The longer a seizure continues, the greater the likelihood of pharmacoresistance to termination and a worse health outcome for the patient, including increased risks of subsequent prolonged seizure activity, memory deficits and learning difficulties [7,8]. In addition, the impact on health resources is greater because these patients require more intensive medical assistance [6],[9-11]. The costs of direct medical care for children with epilepsy can be very high – a recent study in Germany, for example, showed that the mean direct medical cost over three months was €1,940 for children with non-drug-resistant seizures and €3,464 for those with drug-resistant seizures [12]. Prompt treatment with rescue medication is an important aspect of care for children experiencing PACS; however, such treatment provides a particular challenge because these seizures occur predominantly in the community setting where rescue medication and trained carers may not always be available.

A recent study by Anderson et al. noted that the ideal drug for the treatment of convulsive seizures in paediatric patients would have the following characteristics [8]:

 A rapid onset of action.

 A broad spectrum of efficacy; that is, not restricted to a particular seizure type or underlying cause.

 A prolonged duration of action.

 Minimal adverse effects.

 A simple and socially acceptable administration route for both patient and caregiver.

 Easy storage and portability.

In most European countries, no clear guidance is available for the treatment of PACS outside of the hospital setting and no clear guidance is provided for caregivers [13,14].

In Western Europe, the treatment pathways for the use of rescue medication in the management of PACS can be broadly categorised into two groups: many continental European countries primarily advocate the use of licensed treatments, whereas a number of other countries primarily employ unlicensed treatments. More specifically, in Denmark, France, Germany, Italy and Spain the mainstay of rescue treatment is rectal diazepam, which was the only licensed treatment for PACS in children until recently. In Switzerland and Germany, there is frequent off-label buccal use of fast dissolving lorazepam tablets (as well as rectal diazepam). Countries that most commonly use unlicensed buccal midazolam include the UK, Sweden and Norway [13]. A significant body of evidence now supports buccal midazolam as an effective and safe first-line therapy for prolonged seizures [8], which avoids the social stigma of rectal administration.

In September 2011, BUCCOLAM was granted a Paediatric-Use Marketing Authorisation (PUMA) by the European Medicines Agency (EMA) [1]. The PUMA initiative aims to ensure that medicines used to treat children are subject to high-quality, ethical research and are appropriately authorised without subjecting the paediatric population to unnecessary clinical trials [15].

In most European countries, BUCCOLAM is indicated for the treatment of PACS in infants, toddlers, children and adolescents (from three months to <18 years). For infants between three and six months of age, treatment should be in a hospital setting where monitoring is possible and resuscitation equipment is available. In Switzerland, BUCCOLAM is indicated for emergency treatment of PACS lasting more than five minutes in children from six months to 18 years. In all European countries, BUCCOLAM must only be used by parents/carers where the patient has been diagnosed to have epilepsy.

This case study of BUCCOLAM provides an informative illustration of how variation in a treatment pathway can lead to different HTA considerations for the same innovative technology.

## Methods

### Modelling the local treatment pathway

Decision analytic models were constructed to assess the cost-effectiveness of BUCCOLAM in seven European countries. The original model was built for an HTA submission in Scotland [16], and was subsequently adapted for use in Wales, Germany, France, Spain, Italy and Switzerland. In the Republic of Ireland, a full decision analytic model was not required for reimbursement purposes, as BUCCOLAM was accepted following a rapid review.

#### Treatment pathway

In the absence of coherent European guidance on the treatment of PACS in the community, the use of rescue medication is determined locally and is subject to substantial variation. In addition to this, information on the effectiveness of the various rescue medications is not available, with only limited information available on their efficacy. This is the result of many products being used unlicensed or off-label, which is a relatively common practice in paediatric medicine throughout Europe [17].

Clinical opinion was derived from Delphi panel surveys and interviews with clinical experts. The numbers and backgrounds of the participating experts for each country are shown in Additional file 1: Table S1. Expert responses were used to determine the local pattern of care in each jurisdiction and to derive model parameter estimates. In general, experts were consistent in their estimates for parameters used to model hospitalisation and healthcare resource use. A greater variation was seen in estimates for events in the community setting, both between clinicians and by country. This was attributed to the fact that clinicians rarely directly observe treatment in the community, resulting in greater uncertainty in their responses. In Scotland, Wales and Spain, expert opinion was supplemented with parent surveys to address this limitation.

Variation was observed in the treatment pathways of different countries, particularly concerning the rescue medication administered by parents and carers following a seizure in the community setting. The established treatment practice involved the use of an unlicensed buccal midazolam preparation in Scotland and Wales, rectal diazepam in Spain and Italy, and a combination of rectal diazepam with other licensed and/or off-label or unlicensed rescue medications in Germany, France and Switzerland. Differences in paramedic and hospital drug use were also seen between countries. The established current pattern of treatment formed the comparator arm of the model for each country and is shown in Table 1.

**Table 1:** Medications currently administered in the community, by ambulance paramedics and in hospital

**Country**	**Carer administration**	**Paramedic administration**	**Hospital administration**	**Source**
**Scotland**	Buccal midazolam 100%	Rectal diazepam 100%	Buccal midazolam 100%	Delphi panel and patient surveys
**Wales**	Buccal midazolam 95%	Rectal diazepam 100%	Buccal midazolam 38%	Delphi panel and patient surveys
	Rectal diazepam 5%		Rectal diazepam 62%	
**Germany**	Rectal diazepam 81%	Rectal diazepam 82%	Rectal diazepam 82%	Delphi panel
	Buccal use of lorazepam tablets 19%	Lorazepam 6%	Lorazepam 6%	
		Clonazepam 6%	Clonazepam 6%	
		Phenytoin 6%	Phenytoin 6%	
**Spain**	Rectal diazepam 100%	Rectal diazepam 100%	Rectal diazepam 100%	Delphi panel and patient surveys
**France**	Rectal diazepam 92%	Rectal diazepam 84%	Rectal diazepam 84%	Delphi panel
		IV clonazepam 10%	IV clonazepam 10%	
	Oral clonazepam 8%			
		Phenytoin 6%	Phenytoin 6%	
**Italy**	Rectal diazepam 100%	Rectal diazepam 10%	Rectal diazepam 65%	Delphi panel
		IV midazolam 90%	IV midazolam 35%	
**Switzerland**	Rectal diazepam 45%	Rectal diazepam 50%	Rectal diazepam 50%	Clinician interviews
	Buccal lorazepam 54%	IV diazepam 6%	IV diazepam 6%	
	Intranasal midazolam <2%	IV lorazepam 43%	IV lorazepam 43%	
		Intranasal midazolam 1%	Intranasal midazolam 1%	
		IV clonazepam <1%	IV clonazepam <1%	
		Phenobarbital <1%	Phenobarbital <1%	

IV = intravenous.

‘Buccal midazolam’ in this table refers to the unlicensed preparation.

Variation was also observed in the treatment practices used by parents and carers (Table 2). In Wales, Germany, France and Switzerland, some parents and carers had instructions from their clinician to administer a second dose of rescue medication if the first dose did not stop the seizure. In Germany, France and Switzerland, patients were not necessarily taken to hospital after an ambulance call-out, with paramedics administering medication and often awaiting results before taking the child to hospital. These differences were incorporated into the models to better define the decision problem for the relevant healthcare system.

**Table 2:** **Key structural characteristics and major treatment pathway differences**^**a**^**of European cost-effectiveness model adaptations**

**Country**	**Model features**
**Scotland**	● Patient simulation for buccal midazolam comparison
	● Parents/carers only give a single dose
	● Taken to hospital if ambulance called
**Wales**	● Patient simulation for buccal midazolam comparison
	● Allows for second doses of treatment under emergency care plans
	● Taken to hospital if ambulance called
	● Chance of inpatient admission is 100% for patients suffering multiple seizures
	● Additional chance of admission to intensive care for patients suffering multiple seizures
**Germany**	● Allows for second doses to be administered
	● Patient may not necessarily be taken to hospital after ambulance call-out
**Spain**	● Parents/carers only give a single dose
	● Taken to hospital if ambulance called
**France**	● Allows for second doses to be administered
	● Patient may not necessarily be taken to hospital after ambulance call-out
**Italy**	● Parents/carers only give a single dose
	● Taken to hospital if ambulance called
**Switzerland**	● Allows for second doses to be administered
	● Patient may not necessarily be taken to hospital after ambulance call-out

^a^ According to clinician consultation.

#### Efficacy

No data that specifically evaluate the efficacy of buccal midazolam in prefilled syringes are currently available. Five studies comparing the efficacy of buccal midazolam with that of rectal diazepam have been conducted [8]. The source of efficacy data used within the models was the study by McIntyre et al. [18]. This is the only published controlled trial comparing buccal midazolam (the intravenous preparation of midazolam hydrochloride, administered into the buccal cavity) with rectal diazepam that was conducted in a European context with a large sample size (n = 219 seizure episodes). This study also reported outcomes directly related to resource use associated with seizures, which is a key contributor to the expected incremental benefit of BUCCOLAM. Although the trial observed a UK cohort, the comparative efficacy of buccal midazolam in reducing the duration of seizures and the probability of repeat seizures is assumed to be applicable to all the countries considered in this article.

The following assumptions were made in order to use the outcomes of the McIntyre publication within the economic model and estimate the chance of a seizure lasting more than ten minutes, the chance of a repeat seizure and the duration of seizures:

 BUCCOLAM (midazolam oromucosal solution) has equal efficacy to IV midazolam as hydrochloride 5 mg/ml administered by the oromucosal route.

 All other formulations of buccal midazolam and intranasal midazolam have equal efficacy to IV midazolam as hydrochloride 5 mg/ml administered by the oromucosal route.

 Model comparators other than buccal and intranasal midazolam share the same efficacy outcomes as rectal diazepam.

These assumptions were based on the advice of clinical experts in several countries. In the case of off-label medications, the assumption of equal efficacy with rectal diazepam was a conservative estimate as clinical experts in several countries suggested that other off-label medications, such as lorazepam tablets administered by the buccal route, were less efficacious than rectal diazepam. This conclusion is supported by pharmacokinetic evidence indicating that midazolam is more likely to achieve therapeutic plasma concentrations than lorazepam [8].

#### Effectiveness

The primary source of effectiveness data used in the models was clinical expert opinion, which was obtained through Delphi panel research conducted in a number of European countries as part of the cost-effectiveness assessment of BUCCOLAM. The Delphi process involved three rounds: the first consisted of a questionnaire and the second consisted of either a second questionnaire or a group face-to-face meeting at which the initial questionnaire results were presented back to all participants and amended or validated; the final round provided an opportunity for comment or revision on the consensus gained in the second round [19].

The Delphi panels reported a number of different expected advantages for BUCCOLAM compared with each of the main treatment alternatives used in the community. They estimated that BUCCOLAM had both an efficacy and an effectiveness advantage over rectal diazepam and off-label buccal use of lorazepam tablets and, in many cases, that it had an effectiveness advantage compared with unlicensed buccal midazolam. This advantage in effectiveness could be attributed to BUCCOLAM being presented in prefilled syringes and, in some countries, to the additional willingness or ability of carers (such as teachers) to administer a licensed product via the oromucosal route, leading to a reduction in ambulance call-outs. The Delphi panels also estimated a reduction in wastage costs with BUCCOLAM, due to its convenient and efficient mode of packaging (BUCCOLAM in four prefilled unit-dose syringes versus unlicensed buccal midazolam for which the most widely used presentation is a single 10 ml bottle with four syringes).

These results are supported by a recent survey completed by a total of 129 healthcare professionals in six European countries, which indicates that the biggest barrier to administering rescue medication to children suffering PACS in the community is fear of legal consequences, either due to the use of unlicensed medication or due to the lack of social acceptability of rectal administration [13].

### Core model structure

#### Decision tree model

**Figure 1:**
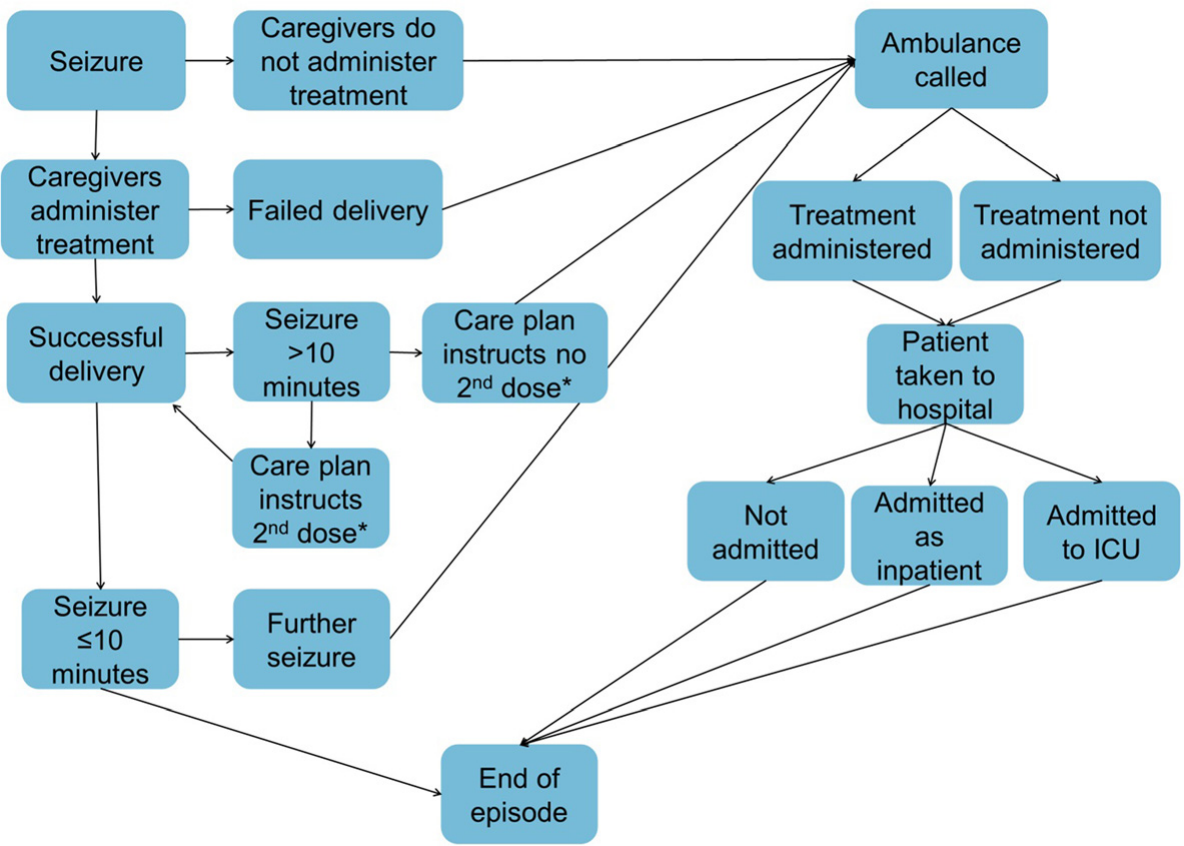
**Structure of decision tree included in all country adaptations [**[16]**].** * 2nd dose must only be given according to prior medical advice.

A decision tree approach was used to reflect the events during and immediately following PACS. The structure of this model, which was built in Microsoft Excel, is shown in Figure 1[16].

A number of events can trigger an ambulance call-out in the decision tree; a carer not administering treatment in the community, failed delivery of treatment by the carer, a seizure that lasts more than ten minutes or a repeat seizure within one hour of the first. Once an ambulance has arrived, paramedics may administer further treatment, and a patient may be taken to hospital. If a patient is taken to hospital, patients may receive further treatment, and may be admitted for a hospital stay.

The decision node probabilities are tailored to accurately represent the pattern of care in each country. The probabilities of seizure cessation, repeat seizures and the duration of seizures are based on the relative efficacy of buccal midazolam compared with rectal diazepam, as reported by McIntyre *et al.*[18]. The parameterisation of these decision nodes for each country is shown in Table 3.

**Table 3:** Node probabilities from the different country adaptations of the model

**Country**	**Chance of carer not administering treatment**	**Chance of failed administration resulting in ambulance call-out**	**Chance of parent/carer administering 2nd dose**	**Chance of inpatient admission after ambulance call-out**	**Chance of ICU admission if admitted to hospital**	**Chance of ambulance taking patient to hospital**^**a**^	**Chance seizure lasts more than 10 minutes**	**Chance of repeat seizure**	**Average number of seizures per month**
**Scotland**	BUCCOLAM 10%; Current care 16%	BUCCOLAM 6%; Current care 8%	BUCCOLAM 0%; Current care 0%	BUCCOLAM 20%; Current care 20%	BUCCOLAM 20%; Current care 20%	–	BUCCOLAM 35%; Current care 35%	BUCCOLAM 14%; Current care 14%	1.26
**Wales**	BUCCOLAM 5%; Current care 6.2%	BUCCOLAM 3.5%; Current care 4.1%	BUCCOLAM 30%; Current care 30%	BUCCOLAM 10%; Current care 10% ^b^	BUCCOLAM 2%; Current care 2%	–	BUCCOLAM 35%; Current care 36%	BUCCOLAM 14%; Current care 33%	1.17
**Germany**	BUCCOLAM 24%; Current care 37%	BUCCOLAM 10%; Current care 18%	BUCCOLAM 10%; Current care 10%	BUCCOLAM 90%; Current care 90%	BUCCOLAM 10%; Current care 10%	BUCCOLAM 90%; Current care 90%	BUCCOLAM 35%; Current care 59%	BUCCOLAM 14%; Current care 33%	1.27
**Spain**	BUCCOLAM 10%; Current Care 70%	BUCCOLAM 5%; Current care 10%	BUCCOLAM 0%; Current care 0%	BUCCOLAM 20%; Current care 66%	BUCCOLAM 20%; Current care 10%	–	BUCCOLAM 35%; Current care 59%	BUCCOLAM 14%; Current care 33%	0.75
**France**	BUCCOLAM 30%; Current Care 50%	BUCCOLAM 10%; Current care 50%	BUCCOLAM 10%; Current care 10%	BUCCOLAM 51%; Current care 51%	BUCCOLAM 23.58%; Current care 23.58%	BUCCOLAM 80%; Current care 80%	BUCCOLAM 35%; Current care 59%	BUCCOLAM 14%; Current care 33%	0.27
**Italy**	BUCCOLAM 30%; Current Care 39%	BUCCOLAM 15%; Current care 40%	BUCCOLAM 0%; Current care 0%	BUCCOLAM 70%; Current care 70%	BUCCOLAM 14%; Current care 14%	–	BUCCOLAM 35%; Current care 59%	BUCCOLAM 14%; Current care 33%	0.37
**Switzerland**	BUCCOLAM 16%; Current care 24%	BUCCOLAM 3%; Current care 14%	BUCCOLAM 20%; Current care 20%	BUCCOLAM 70%; Current care 70%	BUCCOLAM 22.22%; Current care 22.22%	BUCCOLAM 98%; Current care 98%	BUCCOLAM 35%; Current care 59%	BUCCOLAM 14%; Current care 33%	0.32

^a^ Germany, France and Switzerland only; ^b^ In the Welsh model, patient who suffered repeat seizures had a 100% probability of hospital admission.

Medication failure can occur in the model for various reasons, including: no treatment being available at the location of the seizure; poor absorption from the site of administration; or incorrect dose measurement. Delphi panel members and local clinicians were asked to estimate how the availability of a licensed preparation might affect the likelihood of treatment administration in the community setting. Experts in all countries agreed that administration of treatment in the event of a seizure would be more likely with BUCCOLAM (Table 3). Similarly, it was expected that the risk of administration errors resulting in under- or overdosing would be reduced with BUCCOLAM compared with many of the treatments comprising current care.

#### Patient simulation

In the UK, a different amount of wastage is expected for licensed and unlicensed buccal midazolam supplied in four-dose bottles. A patient simulation model was, therefore, incorporated into the UK model to predict the pattern of patients’ seizures and whether or not rescue medication is available at the time of seizure, allowing the calculation of the cost of expected drug wastage over the time horizon of the model. Further details on the simulation model used in the UK cost-effectiveness analysis can be found in Lee et al. [16].

### Costs and resource use

Cost data were typically acquired from national reference price lists for each country, and expert opinion on the expected resource use following PACS was also obtained. The key resource use and drug costs included in the models are shown in Table 4. These are all shown in euros, for ease of comparison.

**Table 4:** Drug and resource costs by country

	**Scotland**	**Wales**	**Germany**	**Spain**	**France**	**Italy**	**Switzerland**
**Reference year**	2012–2013	2012–2013	2012–2013	2012–2013	2011–2012	2012	2012
**Cost elements**							
Ambulance cost	€317.23	€284.68	€0.00 ^a^	€309.36	€1,120.76	€113.27	€1,217.06
A&E: admitted patients	€141.35	€141.35	€542.87	€129.23	–	€335.12	–
A&E: non-admitted patients	€113.41	€113.41	€542.87	€111.10	€25.32	€335.12	€6,194.85
Inpatient admission	€680.16	€680.16	€403.00	€1,616.89	€1,436.21	€1,147.75	€6,194.85
ICU admission	€1,574.28	€1,574.28	€1,115.00	€2,254.91	€8,980.05	€4,322.30	€15,157.31
Drug costs (per dose)							
BUCCOLAM ^b^	€26.97 ^c^	€26.97 ^c^	€28.54	€17.38	€17.17	€22.65	€39.93 ^d^
Rectal diazepam	€2.24	€2.24	€5.08	€0.94	€0.53	€0.00 ^e^	€5.27
Unlicensed buccal midazolam	€30.38 ^f^	€30.66^g^	–	–	–	–	–
Phenytoin	–	–	€6.60	–	€0.06	–	–
Oral clonazepam	–	–	–	–	€0.08	–	–
IV clonazepam	–	–	€1.70	–	€0.79	–	€5.29
Lorazepam tablets	–	–	€0.39	–	–	–	€0.55
IV midazolam	–	–	–	–	–	€4.18	–
Intranasal midazolam	–	–	–	–	–	–	€36.51
Chloral hydrate	–	–	–	–	–	–	€1.40
Phenobarbital	–	–	–	–	–	–	€82.78
IV diazepam	–	–	–	–	–	–	€7.20
IV lorazepam	–	–	–	–	–	–	€2.40

A&E = accident and emergency; ICU = intensive care unit; IV = intravenous.

^a^ Ambulance costs for Germany set to zero as they are included in aggregated A&E costs; ^b^ The drug costs used for BUCCOLAM were defined by local HTA body requirements (e.g. ex factory, public price) and include VAT as appropriate, therefore are not comparable between countries; ^c^ The drug cost used represents an average of each of the four strengths available; ^d^ At the time of publication, the approved price for BUCCOLAM in this country has not been agreed – the price shown is the maximum price allowable under the price referencing rules in Switzerland.

^e^ Italian cost of rectal diazepam is zero as this drug is not reimbursed by the Italian national health service; ^f^ Based on May 2013 Drug Tariff [20]; ^g^ Based on May 2013 Drug Tariff price plus a sourcing fee for medicines classed as ‘specials’ [21,22].

All costs are presented in Euros, converted on 30/10/12, using the following rates from xe.com currency converter: 1 Euro (EUR) = 0.806579 British Pound (GBP), 0.82165 Swiss Francs (CHF).

The price of BUCCOLAM included in each adaptation of the model was current at the time it was developed. UK costs have been updated to the most recent cost data and so differ from those quoted in the HTA submissions in Scotland and Wales. As the price for BUCCOLAM in Switzerland has not yet been determined, the price used in the model is the maximum price allowable under the price referencing rules in Switzerland and is based on the agreed prices in the reference countries.

### Health-related quality of life

The quality-adjusted life-year (QALY) decrement associated with PACS was calculated as the product of the duration of a seizure event and the estimated health-related utility value during this time. The former was broken down to represent three phases of a seizure episode:

 During the seizure

 Postictal period – the period during which the brain recovers immediately following the seizure

 Recovery period – the time taken for the patient to recover back to baseline utility.

Due to a paucity of published utility data pertaining to a child’s health status during a seizure episode, health-related quality of life (HRQoL) estimates were elicited from clinical experts The utility values used in the analyses were initially measured for the construction of the Scottish model. These values were based on the estimates of four UK clinicians, who had been asked to complete a five-level EuroQol five dimensions (EQ-5D-5L) questionnaire from the perspective of a child suffering a seizure. It is neither feasible nor ethical to administer a patient reported outcome measure to a child during or shortly after a seizure. Therefore the methods established as best practice for capturing HRQoL for *chronic* conditions would have been unsuitable for estimating the effect of *acute* episodes on HRQoL.

Repeating the measurement and valuation exercises for each country was not feasible; especially as EQ-5D-5L tariffs were not available for all the countries considered. To include the appropriate perspective in valuing the health impact of the intervention for each country, the UK values were distributed to the local Delphi panel members and key opinion leaders (KOLs) to adjust and validate based on their own expertise (Table 5).

**Table 5:** Utility values and event durations used to calculate QALYs lost to seizures

**Utility values**	**Scotland**	**Wales**	**Germany**	**Spain**	**France**	**Italy**	**Switzerland**
**Proportion of patients with severe disability**	10%	20%	20%	10%	46%	50%	17.5%
**Quality of life for patients without severe disabilities**							
Baseline	0.879	0.920	0.920	0.879	0.879	0.920	0.864
During seizure	−0.204	−0.334	−0.334	−0.204	−0.436	−0.334	−0.436
Following seizure – no ambulance	0.722	0.722	0.722	0.722	0.507	0.722	0.358
Following seizure –ambulance called	0.100	0.100	0.100	0.100	0.413	0.100	0.336
**Quality of life for patients with severe disabilities**							
Baseline	−0.127	−0.127	−0.127	−0.127	−0.001	−0.127	−0.127
During seizure	−0.359	−0.359	−0.359	−0.359	−0.516	−0.359	−0.594
Following seizure – no ambulance	−0.313	−0.313	−0.313	−0.313	−0.230	−0.313	−0.313
Following seizure – ambulance called	−0.313	−0.313	−0.313	−0.313	−0.216	−0.313	−0.313
**Event timings**							
Average duration of seizure (minutes)	BUCCOLAM 8; Current care 8	BUCCOLAM 8; Current care 8.35	BUCCOLAM 8; Current care 15	BUCCOLAM 8; Current care 15	BUCCOLAM 8; Current care 15	BUCCOLAM 8; Current care 15	BUCCOLAM 8; Current care 15
**Time to recovery (hours) – where available, the duration of the postictal phase is shown in parentheses**							
No ambulance	21.0	24.0 (4.0)	0.9 (0.6)	21.0	1.25 (0.2)	21.0	BUCCOLAM 1.25; Current care 1.24 (0.2)
Ambulance but no hospitalisation	40.5	30.0 (4.0)	3.0 (1.0)	40.5	2.0 (0.6)	40.5	BUCCOLAM 2.00; Current care 1.98 (0.6)
Ambulance and hospitalisation	64.5	96.0 (14.0)	6.0 (2.5)	64.5	12.5 (0.6)	112.5	BUCCOLAM 12.50; Current care 12.48 (0.6)
Ambulance and hospitalisation in intensive care unit	88.5	120.0 (24.0)	6.0 (2.5)	88.5	12.5 (0.6)	136.5	BUCCOLAM 12.50; Current care 12.48 (0.6)

QALYs = quality-adjusted life-years.

The average time taken to recover to baseline HRQoL differed according to the events that followed the seizure. Post-seizure events were categorised into the following four scenarios:

 There was no need for an ambulance to be called

 An ambulance was called, but the patient was not admitted to hospital

 The patient was admitted to hospital, but not into an intensive care unit (ICU)

 The patient was admitted to an ICU.

Following the end of a seizure, it is assumed that the recovery of utility at the start of the postictal period is instant, and the recovery back to baseline utility is linear over the duration of the recovery period.

The duration of the seizure itself was determined within the model by the treatment received and was based on the publication by McIntyre et al. [18]: eight minutes for seizures treated with BUCCOLAM, or buccal or intranasal midazolam, and 15 minutes for rectal diazepam or other rescue medications. The duration of the postictal and recovery periods in each of these scenarios was estimated by the Delphi panel members and clinicians in each country, to accurately represent the expected HRQoL impact on the patient.

Both the HRQoL estimates and the expected duration of seizure events are summarised by country in Table 5. An additional consideration highlighted by clinical experts was a subpopulation of children who suffer from a severe disability as a result of their epilepsy; for these children, the QALY loss due to a seizure was expected to be much lower because of their lower baseline HRQoL. The proportion of patients estimated to have a severe disability in each country is reported in Table 5, together with the reduced baseline and event utility implemented for these patients.

### Model outputs

The primary outputs of the models were the incremental costs and QALYs associated with implementation of the licensed BUCCOLAM preparation. All country models featured an estimate of the expected annual budget impact associated with adopting BUCCOLAM as standard care. These analyses were informed by national registry data and estimates provided by local Delphi panel members and KOLs.

Results presented within this manuscript are for an average year, with no discounting being applied.

#### Deterministic sensitivity analyses

Deterministic sensitivity analyses were performed to assess the robustness of the models to changes in key parameter values. The upper and lower bounds of each parameter were modelled to calculate the maximum and minimum incremental cost-effectiveness ratios (ICERs) that can be produced by each parameter independently. These analyses assess the sensitivity of the models to the parameter estimates elicited from clinicians and Delphi panel members, and estimate the direct impact of treatment pathway characteristics on the cost-effectiveness of BUCCOLAM across countries.

## Results

### Base case results

The base case analysis showed that BUCCOLAM dominated current care, rectal diazepam and unlicensed buccal midazolam in all countries. This dominance over current care indicates that BUCCOLAM is both less costly and more beneficial for patients in all of the countries included in the analyses (Table 6).

**Table 6:** Base case results by country, one-year time horizon

**Country**	**Current care**			**Rectal diazepam**			**Unlicensed buccal midazolam**			**BUCCOLAM**		
	**Costs**	**QALYs**	**No of ambulance call-outs**	**Costs**	**QALYs**	**No of ambulance call-outs**	**Costs**	**QALYs**	**No of ambulance call-outs**	**Costs**	**QALYs**	**No of ambulance call-outs**
**Scotland**	€5,544	0.75030	8.31	€6,997	0.74475	11.45	€5,544	0.75030	8.31	€5,145	0.75112	7.83
**Wales**	€5,019	0.68030	6.23	€7,539	0.67016	9.65	€4,565	0.68231	6.05	€4,412	0.68458	5.87
**Germany**	€13,278	0.69522	12.99	€13,340	0.69520	13.04	N/A			€ 9,770	0.69662	9.20
**Spain**	€12,862	0.74736	8.34	€12,862	0.74736	8.34				€7,378	0.75543	4.69
**France**	€8,550	0.47491	3.02	€8,550	0.47491	3.02				€5,913	0.47516	2.07
**Italy**	€6,214	0.38552	3.96	€6,214	0.38552	3.96				€4,674	0.38808	2.92
**Switzerland**	€27,174	0.68921	2.99	€28,588	0.68916	3.15				€17,777	0.68957	1.94
**Country**	**Incremental versus current care**			**Incremental versus rectal diazepam**					**Incremental versus unlicensed buccal midazolam**			
	**Costs**	**QALYs**	**Ambulance call-outs avoided**	**ICER**	**Costs**	**QALYs**	**Ambulance call-outs avoided**	**ICER**	**Costs**	**QALYs**	**Ambulance call-outs avoided**	**ICER**
**Scotland**	–€399	0.00082	0.48	Dominant ^a^	–€1,852	0.00637	3.62	Dominant ^a^	–€399	0.00082	0.48	Dominant ^a^
**Wales**	–€607	0.00429	0.36	Dominant ^a^	–€3,127	0.01442	3.79	Dominant ^a^	–€153	0.00227	0.18	Dominant ^a^
**Germany**	–€3,507	0.00140	3.79	Dominant ^a^	–€3,569	0.00142	3.84	Dominant ^a^	N/A			
**Spain**	–€5,484	0.00807	3.64	Dominant ^a^	–€5,484	0.00807	3.64	Dominant ^a^				
**France**	–€2,637	0.00025	0.95	Dominant ^a^	–€2,637	0.00025	0.95	Dominant ^a^				
**Italy**	–€1,540	0.00256	1.03	Dominant ^a^	–€1,540	0.00256	1.03	Dominant ^a^				
**Switzerland**	–€9,397	0.00036	1.05	Dominant ^a^	–€10,811	0.00041	1.21	Dominant ^a^				

ICER = incremental cost-effectiveness ratio; N/A = not applicable as unlicensed buccal midazolam is not used in these countries; QALY = quality-adjusted life-year.

^a^ Dominant means that BUCCOLAM is both cost saving and results in positive outcomes when compared with the relevant comparator.

Substantial variation in the expected cost savings resulting from treatment with BUCCOLAM was observed between the different countries. The model for Scotland resulted in the lowest predicted cost saving and the smallest expected impact of BUCCOLAM compared with the unlicensed preparation. The Swiss model predicted the largest cost saving following the introduction of BUCCOLAM. This large saving has multiple causes: first, the clinical experts in Switzerland predicted a large difference in effectiveness between BUCCOLAM and rectal diazepam, due to carer unwillingness to administer rectal treatments and the ease of delivery of BUCCOLAM; second, the resource use costs associated with ambulance call-outs and hospitalisations were greater than those for the other countries considered in the analyses (Table 4); finally, a comparatively high proportion of individuals experiencing treatment failure were expected to be admitted as inpatients or to an ICU. In countries where unlicensed buccal midazolam is the comparator (Scotland and Wales), the overall cost savings were lower than in countries that typically use other rescue medications.

A breakdown of the incremental costs and savings of adopting BUCCOLAM over the current pattern of treatment is shown in Table 7. The ambulance costs and A&E costs for patients who were not admitted to hospital were zero in both arms of the German model. The first is because ambulance costs are included in the costs of an A&E visit, and the second because clinical experts clarified that patients would always be admitted following an A&E visit, either as an inpatient or to an ICU.

**Table 7:** Breakdown of base case incremental costs of BUCCOLAM versus standard care, one-year time horizon

**Costs**	**Scotland**	**Wales**	**Germany**	**Spain**	**France**	**Italy**	**Switzerland**
Drug costs	–€107.86	–€15.77	€342.99	€133.09	€43.85	€81.03	€138.07
Ambulance costs	–€152.05	–€101.44	€0.00	–€1,127.20	-€1,061.04	–€116.98	–€1,279.31
A&E costs (no admission)	–€43.48	€29.73	€0.00	–€137.64	-€6.47	–€103.83	-€640.31
Inpatient admission costs	–€63.00	–€497.96	–€3,222.80	–€3,779.18	-€550.67	–€918.89	–€4,482.20
ICU admission costs	–€32.89	–€21.22	–€627.64	–€573.34	-€1,062.69	–€481.01	–€3,133.39
Total	–€399.28	–€606.66	–€3,507.45	–€5,484.26	-€2,637.02	–€1,539.68	–€9,397.13

A&E = accident and emergency; ICU = intensive care unit.

### Source of incremental benefit

The cost differences associated with adopting BUCCOLAM, a licensed oromucosal midazolam preparation, are dependent on the standard treatment pathway in each country, especially the type of rescue medication used.

Where the comparator is either rectal diazepam or bucally administered lorazepam tablets, the cost differences when adopting BUCCOLAM are due to:

 An increase in drug purchase costs compared with rectal diazepam and bucally administered lorazepam tablets

 Increased social acceptability – with some countries also having legislation preventing the administration of rectal treatments to children by carers (such as teachers) – leading to a greater willingness/ability to administer treatment in the community setting compared with rectal diazepam

 The availability of a licensed treatment, leading to a greater willingness/ability to administer treatment and increased confidence in use in the community setting compared with bucally administered lorazepam tablets

 A reduction in the likelihood of failed administration due to a simpler treatment process compared with rectal diazepam

 Increased efficacy in stopping seizures and preventing repeat seizures that result in ambulance call-out compared with rectal diazepam and bucally administered lorazepam tablets

 A reduction in the duration of seizures and associated utility decrement compared with rectal diazepam and bucally administered lorazepam tablets

Where the comparator is unlicensed buccal midazolam, the cost differences when adopting BUCCOLAM are due to:

 A reduction in drug costs compared with unlicensed product tariffs

 Avoidance of drug wastage due to the supply in prefilled unit-dose syringes rather than stock bottles, which were reported as the most widely used presentation of unlicensed buccal midazolam

 A greater willingness and/or ability of parents and carers to administer treatment in the community setting due to increased accessibility of rescue medication at multiple locations

 A reduction in the likelihood of failed administration due to greater dosing accuracy

By increasing the proportion of patients in the more favourable decision tree outcomes (that is, avoidance of ambulance call-outs and hospital admissions), BUCCOLAM is expected to reduce the resource use and HRQoL burdens of PACS, driving the incremental cost-effectiveness over standard care in all countries.

### Budget impact

Table 8 shows an expected budget impact between €840,808 and €65.8 million per country for the first year following the introduction of BUCCOLAM.

**Table 8:** The expected annual healthcare budget impact of adopting BUCCOLAM

	**Scotland**	**Wales**	**Germany**	**Spain**	**France**	**Italy**	**Switzerland**
Population aged 0–18 years		2,993,000	12,662,656		15,226,230	10,831,152	1,469,605
Prevalence rate			0.45%		0.50%	0.75%	0.45%
Absolute prevalence			57,558	100,000	76,131	81,234	6,680
Proportion receiving rescue medication			48.0%		30.0%		96.7%
Absolute number receiving rescue medication		2,737	27,628		22,839		6,457
Expected one-year mortality ^a^		0.02%					
Patients alive to receive treatment ^a^		2,736					
Proportion eligible to receive BUCCOLAM			70%	25%	110% ^b^		80%
Number of patients eligible	6,820		19,339	25,000	25,123		5,166
Market share in first year	50.0%	50.0%	10.0%	48%	17.9%	10.0%	36.7%
Number of patients transferring from current care to BUCCOLAM	3,410	1,368	1,934	12,000	4,504	8,123	1,894
**Incremental saving per patient**^**c**^	**€399**	**€607**	**€3,507**	**€5,484**	**€2,637**	**€1,540**	**€9,397**
**Total expected saving**^**d**^	**€1,361,549**	**€830,203**	**€6,783,169**	**€65,811,104**	**€11,877,804**	**€12,507,399**	**€17,801,274**

^a^ Information on mortality is required in the Welsh health technology assessment submission template, and this was provided in addition to the base case calculation of the budget impact of BUCCOLAM. For other countries, this was not included, as mortality rates are low and not expected to be substantially impacted by the rescue medication given.

^b^ In France, it was expected that the number of children being prescribed rescue medication would increase over current levels with the availability of BUCCOLAM.

^c^ The incremental saving per patient is taken from Table 7 (Breakdown of base case incremental costs of BUCCOLAM versus standard care, one-year time horizon).

^d^ The total expected saving is calculated as the product of the incremental saving per patient and the number of patients transferring from current care to BUCCOLAM.

National epidemiology sources were used to estimate the patient population; that is, the prevailing number of epilepsy patients who suffer breakthrough seizures and are eligible to receive rescue medication, as well as the expected number of incident patients each year. Among this patient population, a given percentage was assumed to take up BUCCOLAM in the first year following its introduction, accruing the savings observed in the model (see Table 8).

The estimated total healthcare budget impact was largely determined by the expected savings per patient, but was also influenced by the market uptake of BUCCOLAM, which was estimated at 10–50% in the first year of availability and was typically expected to rise in subsequent years.

### Sensitivity analyses

#### Deterministic sensitivity analysis

**Figure 2:**
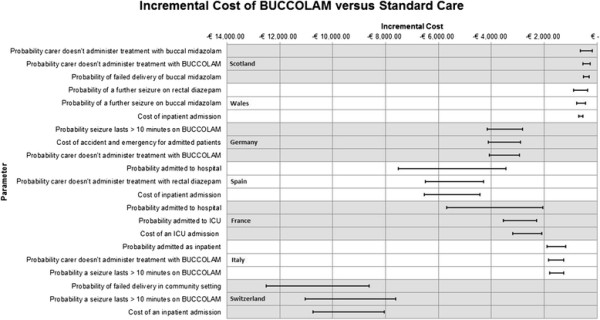
Impact of the three most influential parameters on incremental cost saving of BUCCOLAM.

Figure 2 shows the impact of the three most influential parameters from each model. BUCCOLAM remained cost saving for all parameters tested. The Swiss adaptation contained the greatest uncertainty in results, but also had the greatest expected cost saving.

A large proportion (12/21) of the most influential parameters was based on estimates from clinical experts and patient surveys. Other influential parameters included unit costs (5/21) and efficacy outcomes (4/21).

## Discussion

When assessing the cost-effectiveness of a healthcare intervention, the potential impact of this intervention can be more accurately predicted by accounting for inconsistencies in the treatment delivery pathways and health service resource use. The aim of this study was to examine the influence of differences in the treatment patterns for PACS on the economic evaluation of BUCCOLAM. The analysis demonstrated a cost saving with the introduction of BUCCOLAM in all the countries evaluated. This was driven by different factors, depending on the pathway in which the drug was evaluated.

The pathway characteristic that most heavily influenced the potential saving achieved with BUCCOLAM was the rescue medication currently used in the treatment of PACS. BUCCOLAM was estimated to provide greater incremental benefit when compared with rectal diazepam (primarily as a result of increased efficacy and social acceptability leading to increased use) than compared with an unlicensed preparation of midazolam (where benefits were limited to drug price differences and more probable and successful administration by carers). This meant that the adoption of BUCCOLAM was of greater value in healthcare systems in which rectal diazepam was standard care.

In addition to the choice of comparator, a number of other pathway characteristics influenced the degree of cost saving. These fell into two categories: the value of reducing healthcare resource use and the procedure by which PACS are treated. The former is evident when considering the variation in unit costs, which is explained by differences in tariffs and other factors, including the average length of stay and the staff time involved; thus, the organisation and delivery of local healthcare resources impact on the magnitude of the incremental cost of adopting BUCCOLAM. The latter is a result of the treatment pathway parameters estimated by the clinical experts. These parameter values are indicative of the treatment provided in each healthcare system; for example, the criteria that determine the requirement for hospital admission differ by country, so that patients with less severe needs might be hospitalised in one country but not in another. These parameters determine the likelihood that treatment with BUCCOLAM will affect the events following a seizure episode and result in the potential savings from reduced resource use. The sensitivity analyses performed show that variation in those parameters heavily impacted the expected savings achieved with BUCCOLAM, with 12 out of the top 21 influential parameters having been sourced from expert opinion or patient surveys.

This highlights the sensitivity of the model to the responses of the Delphi panel and survey participants. Firstly, it demonstrates how the differences between the countries impact the outcomes of the model. Secondly, it illustrates that the method used to elicit parameter values from clinicians introduces considerable uncertainty into the analyses. However, the results of the deterministic analysis suggest that this does not significantly change the cost-effectiveness outcomes for BUCCOLAM in the decision problem considered here (Figure 2).

A patient’s needs upon hospitalisation are likely to further affect the unit costs used to model the treatment of the average patient (less severely ill patients who do not require as much medical attention will bring down the average cost of a hospital stay). The provision of care in each system, therefore, directly influences both the likelihood and the potential value of successfully treating a seizure episode in the community and avoiding referral to secondary care services.

A number of issues, inherent with the episodic nature of the disease, presented themselves during the construction of the models. While the direct impact of rescue medications on stopping a seizure episode can be observed in a clinical setting (for example, in the study by McIntyre et al.) [18], the events relevant to the decision problem take place in the community setting. This means that obtaining unbiased estimates of how these events might be influenced by the choice of treatment is problematic; for example, the sequence of events is expected to differ according to the cultural context in which the seizure takes place. These societal aspects of healthcare provision include the daily routine of the child, the level of training offered to parents and teachers, and the inclination (or disinclination) of caregivers to perform rectal administration. Capturing these factors accurately in an economic model requires a level of detail that is difficult to achieve through traditional data collection methods, particularly given the known high levels of country-to-country variation in aspects such as carer training and willingness to administer rescue treatments in the community [13,14]. Where parameter values have been estimated by proxy, model outcomes can be confirmed through incorporation of primary observational data, this work is ongoing as part of the PERFECT study [13,14].

Another limitation when constructing the models was the difficulty in obtaining HRQoL values to estimate the effect of a seizure episode on utility. There will be some error introduced by the indirect nature of the estimation of utility values, but it is not clear whether this will under- or over-estimate patient utility. Although there is a consensus in published comparisons of utility estimates from patients and proxies that indirect estimates must be interpreted with caution, the direction of the error is not consistent [23-25]. Although the derivation of utility estimates is not ideal, the particular characteristics of this condition and currently available instruments mean that it is the most appropriate option. As there is no way of directly measuring utility during a seizure episode, the only method of eliciting utilities is by proxy, either from clinicians or parents. Consequently, the EQ-5D (designed for people older than 12 years) rather than the youth version (EQ-5D-Y – designed for children aged between five and 12 years [26]) has been used. The only difference in the versions is that the wording in the youth version has been adapted for younger readers. Furthermore, standard HRQoL instruments are designed to evaluate chronic health states and not to capture the HRQoL impact of acute, short-lived episodes, and it is the QALY loss during seizure episodes that is important in considering the treatment effect of BUCCOLAM.

Sensitivity analyses examining the influence of utility estimates suggest that the absolute utility value does not affect the overall outcome of the model. The incremental QALY gain with BUCCOLAM is derived from the reduced utility decrements associated with the shortened seizures and recovery times. If the treatment is demonstrated to reduce the duration and ultimate severity of the seizure, then the change in QALYs will always be positive (or zero), regardless of the absolute utility baseline and the magnitude of the decrement. It may be that this QALY gain is negligible, but this is simply due to the short-lived nature of the events and the quality of life benefit to the patient may be under-represented in traditional QALY calculations.

The patient population in which BUCCOLAM is indicated presents additional obstacles. A common problem associated with the economic evaluation of paediatric interventions is the lack of availability of relevant clinical trial data, due to unwillingness to recruit children into clinical trials. This results in a reliance on off-label drug formulations in clinical practice [14]. The problem is compounded by the authorisation of BUCCOLAM by the PUMA process, which is designed, in part, to reduce unnecessary enrolment of children to clinical trials and reduce the requirement for paediatric-specific data to obtain a licence. This can result in a paucity of data for the purposes of reimbursement decisions for the intended patient population.

The limitations discussed in this section resulted in a greater reliance on expert opinion, rather than on published, peer-reviewed evidence, to construct the models. This presents a number of technical issues; although clinicians may provide point estimates of key model parameters, they are much less able to make probabilistic statements regarding the estimates to be used in sensitivity analyses. The requirement for arbitrary variation and bounds means that, although probabilistic analysis can be undertaken, this does not capture fully the structural uncertainty underlying the model. In addition, the process used to capture expert opinion was more stringent in some countries than others, with the numbers of clinicians consulted ranging from four to ten per country. The results for Switzerland in particular are limited due to a low number of participating clinicians and an inability to apply a full Delphi process over three rounds. However, the consistency of the cost-saving result with respect to the adoption of BUCCOLAM across the range of countries evaluated suggests that the results may be generalisable to other countries within Europe.

The total cost of managing epilepsy was estimated at €15.5 billion across the 25 EU countries in 2004; this included both direct and indirect costs [27]. Rescue medication accounted for only €400 million (approximately 2.5%) of this total cost, and around 15% of the direct healthcare costs (removing indirect and non-medical costs) for epilepsy. As the cost of rescue medication is comparatively small in relation to the overall burden of managing epilepsy, determining optimal treatment pathways for PACS is considered a low priority for appraisal bodies. This is evident in the decision by the National Institute for Health and Clinical Excellence not to refer BUCCOLAM for a Single Technology Appraisal.

This manuscript highlights, however, that although the price and target population of products such as BUCCOLAM may not be considered significant enough to warrant a full review of the drug’s cost-effectiveness, the cost saving impact on the healthcare systems (and therefore on budgets) can be substantial.

## Conclusions

The results of this evaluation demonstrate that in the treatment of PACS in the community setting, BUCCOLAM is dominant (reduced costs and greater QALYs) over current care in each of the countries modelled. This is the case despite differences in the patterns of care, local costs and substantial data limitations. Comprehensive sensitivity analyses show that model outputs are robust for all countries.

A key conclusion of the evidence generated for the model was that the availability of BUCCOLAM prefilled syringes is likely to increase both the willingness and ability of parents and carers to administer treatment in the community. This improves the effectiveness of the treatment of PACS, resulting in better outcomes compared with rectal diazepam. As a result of these improved outcomes, BUCCOLAM was estimated to consistently reduce ambulance call-out and hospitalisation rates for the treatment of PACS across a range of healthcare systems.

The capacity for BUCCOLAM to reduce resource use and expenditure following a seizure episode has been demonstrated across a range of European healthcare systems. Although the expected savings may be considered small in relation to the total international economic burden of treating epilepsy, this does not diminish the improved outcomes experienced by the individual and the substantial savings that can be achieved compared with current care.

## Competing interests

This study was funded by ViroPharma SPRL-BVBA. ViroPharma is the manufacturer of BUCCOLAM® (midazolam oromucosal solution). The authors independently conducted all analyses and wrote the manuscript. The authors controlled the decision to write and submit the manuscript for publication.

## Authors’ contributions

The study was designed by DL, AH, NB, AS and ET. The methods were implemented by DL, DG, JP and AH. DL, DG, AH, NB, AS and ET participated in the review and interpretation of the data analysis. Delphi Panels were conducted by AH and NB. The manuscript was written by DL, JP and DG, and reviewed by AH, AS, NB and ET. DL is the guarantor for the overall content of this paper. All authors read and approved the final manuscript.

## Additional file

## Supplementary Material

Additional file 1: Table S1. Delphi participants by country. Click here for file
