# Supplementary material for: A comparison of the cost-effectiveness of treatment of prolonged acute convulsive epileptic seizures in children across Europe
Source: Health Econ Rev. 2014 Apr 12;4:6. doi: 10.1186/s13561-014-0006-6 (PMC4052771; doi:10.1186/s13561-014-0006-6)
Supplement: Additional file 1: Table S1. — Delphi participants by country. [file s13561-014-0006-6-S1.doc]

Table S1. Delphi participants by country

| **Country** | **Delphi panellist/ Key opinion leader 1** | **Delphi panellist/ Key opinion leader 2** | **Delphi panellist/ Key opinion leader 3** | **Delphi panellist/ Key opinion leader 4** | **Delphi panellist/ Key opinion leader 5** | **Delphi panellist/ Key opinion leader 6** | **Delphi panellist/ Key opinion leader 7** | **Delphi panellist/ Key opinion leader 8** | **Delphi panellist/ Key opinion leader 9** | **Delphi panellist/ Key opinion leader 10** | **Delphi panellist/ Key opinion leader 11** |
| --- | --- | --- | --- | --- | --- | --- | --- | --- | --- | --- | --- |
| **Scotland** | Consultant paediatrician. Interest in training non-medical persons in community settings to administer rescue medications | Nurse specialist in paediatric epilepsy, experienced in buccal and nasal midazolam. Involved in training and guideline development | Consultant paediatric neurologist with interest in epilepsy. Published author in the subject area | Neonatal and paediatric neurosurgical pharmacist | Consultant child neurologist |  |  |  |  |  |  |
| **Wales** | Consultant paediatric neurologist providing specialist services to children in South East Wales | Clinical nurse specialist in epilepsy. Responsible for training focusing on acute seizures and rescue medication | Senior university lecturer in clinical neurology and neurosciences in North Wales | Consultant in emergency medicine working in a large department in South Wales | Lead pharmacist in neurosciences/ education and training at children’s foundation trust |  |  |  |  |  |  |
| **Germany** | Head of Paediatric Department of a tertiary Epilepsy centre in Southern Germany | Head of a Northern German Epilepsy Centre for children | Physician in a hospital epilepsy unit | Senior consultant at one of the largest epilepsy centers in Germany | Head of a university child neurology department in mid Germany |  |  |  |  |  |  |
| **Spain** | Neuro-paediatrician at a hospital in Madrid | Neuro-paediatrician at a hospital in Madrid | Neuro-physiologist at a hospital in Valencia | Neuro-paediatrician at a hospital in Toledo | Neuro-paediatrician and neurophysiologist at a hospital in Barcelona | Neuro-paediatrician at a hospital in Barcelona | Neuro-paediatrician at a hospital in Córdoba | Neuro-paediatrician at a university hospital in Salamanca | Neuro-paediatrician at a hospital in Mallorca | Neuro-paediatrician at a hospital in Vigo | Neuro-paediatrician at a university clinic in Pamplona |
| **France** | Clinician responsible for an epilepsy unit working almost exclusively with children | Paediatric neurologist in Paris with strong experience in the emergency treatment of PACS in children and adolescents. | Clinician in south west France working on the child neurology unit 20% of the time. | Clinician at a university hospital and regional hospital receiving epileptic children from Basse-Normandie region. | Child neurologist in a general hospital in northern France and a Director of a medico-social centre. | Head of the child neurology department in a Hospital, taking care of children and adolescents with PACS. | Head of a paediatric neurology unit in northern France. | Consultant in child neurology unit in north Paris. |  |  |  |
| **Italy** | Director of an Epilepsy Center.  Author of guidelines on Convulsive Status Epileptics in children. | Neurologist, with experience in epilepsy in adults. Researcher in the field of epilepsy. | Director of an Epilepsy Center, Coordinator of clinics for epileptic patients and clinics for paediatric epilepsy. | Director of the Paediatric Neuropsychiatry Unit. Daily consultancy activity for Paediatric ER. | Director of the Department of Neurosciences and Neuro-rehabilitation.  Center for the treatment of epilepsy. | Associate Professor of Paediatrics. Responsible for the University Center of Paediatric Neurology. | Director of the Center for the diagnosis and treatment of epilepsy. | Director of Complex Unit in a hospital with both general and paediatric ER. |  |  |  |
| **Switz-erland** | Neuropaediatric consultant; Also on regular duty in paediatric emergency department. | Head of Division of Neuropaediatrics and Developmental Medicine in a Children’s University Hospital. | Medical Director of the Swiss epilepsy centre. Adult neurologist. | Senior paediatric neurologist and epileptologist responsible for the special consultation of epileptology and sleep at Children’s Hospital. |  |  |  |  |  |  |  |
